# The Role of Plasmids in the Multiple Antibiotic Resistance Transfer in ESBLs-Producing *Escherichia coli* Isolated From Wastewater Treatment Plants

**DOI:** 10.3389/fmicb.2019.00633

**Published:** 2019-04-03

**Authors:** Qing Li, Weishan Chang, Hongna Zhang, Dong Hu, Xuepeng Wang

**Affiliations:** ^1^Shandong Provincial Key Laboratory of Animal Biotechnology and Disease Control and Prevention, Shandong Agricultural University, Tai’an, China; ^2^Laboratory for Marine Fisheries Science and Food Production Processes, Qingdao National Laboratory for Marine Science and Technology, Qingdao, China; ^3^Department of Teaching Affairs, Hebei University of Economics and Business, Shijiazhuang, China; ^4^Shandong Provincial Engineering Technology Research Center of Animal Disease Control and Prevention, Shandong Agricultural University, Tai’an, China

**Keywords:** *Escherichia coli*, ESBLs, multiple antibiotic resistant, transconjugants, plasmid

## Abstract

We compared the diversity of extended-spectrum β-lactamases (ESBLs) producing *Escherichia coli* (*E. coli*) in wastewater of a municipal wastewater treatment plant. This was done by analyzing multiple antibiotic resistant phenotypes and genotypes. Also, we investigated the antibiotic resistance transfer mechanism of the plasmid by comparing the antibiotic resistance gene linked transfer using a conjugative test, and by analyzing the full-length DNA sequence of one plasmid. The results showed that 50 ESBLs-producing *E. coli* isolates were isolated from 80 wastewater samples at the rate of 62.5% (50/80), out of which 35 transconjugants were obtained with the multiple antibiotic resistant transfer rate as high as 70.0% (35/50). Multiple antibiotic resistance was shown in all transconjugants and donor bacteria, which were capable of resistance to 11 out of 15 kinds of antibiotics. Both transconjugants and donors were capable of resistance to the Ampicillin and Cefalotin at a rate of 100.00% (35/35), while the total antibiotic resistant spectrum of transconjugants narrowed at the rate of 94.29% (33/35) and broadened at the rate of 5.71% (2/35) after conjugate to the donor bacteria. PCR showed that the resistant genotypes decreased or remained unchanged when compared to donor bacteria with transconjugants while the *bla_TEM_* and *bla_CTX-M_* genes were transferred and linked at a rate of 100.00% (35/35) and the *bla_SHV_* gene was at the rate as high as 94.29% (33/35). However, the *qnrS* gene was transferred at a low rate of 4.17% (1/24). In addition, the major resistance gene subtypes were *bla_TEM-_*_1_, *bla_SHV -11_*, and *bla_CTX-M-15_* according to sequencing and Blast comparison. Plasmid wwA8 is a closed-loop DNA molecule with 83157 bp, and contains 45 predicted genes, including three antibiotic resistant resistance genes, *bla_CTX-M-15_*, *bla_TEM-1_* and *qnrS1*, which can be transferred with *E. coli in vitro*. This study shows that *E. coli* isolated from wastewater was capable of transferring resistance genes and producing antibiotic resistant phenotypes. The plasmids containing different resistance genes in *E. coli* play an important role in the multiple antibiotic resistant transfer. Most importantly, antibiotic resistant resistance genes have different transfer efficiencies, the *bla_TEM_* and *bla_CTX-M_* genes transferred at a rate of 100.00% and linked transfer in all 35 transconjugants.

## Introduction

Enterobacteriaceae, particularly *Escherichia coli* (*E. coli*), are among the most important zoonotic pathogens. They are widely distributed in aquatic environments and can cause infectious disease in most animals and humans, such as urinary tract infections, diarrhoea, enteritis, and septicaemia (Lewis et al., 2007; Ang et al., 2016). Abuse and overuse of antibiotics in the clinic has resulted in the emergence of multiple antibiotic resistant bacteria strains (Goldman, 2004). In addition, an increase in the prevalence of multiple antibiotic resistant *E. coli* isolates has been reported worldwide. In recent decades, beta-lactams, as well as fluoroquinolones have been used as important therapeutic choices against bacterial infection. Therefore, the selective pressure resulting from their use and sometimes misuse contributes to antibiotic resistance (Ben Said et al., 2016; Correia et al., 2016). One of the most important mechanisms is the plasmid-mediated production of extended-spectrum β-lactamases (ESBLs), which can hydrolyze β-lactams (Ramos et al., 2013). ESBLs is a group of enzymes that can hydrolyze penicillin and also can hydrolyze the first, second, and third generations of antibiotics, such as Cephalosporins and Aztreonam. ESBLs can be inhibited by enzyme inhibitors, which are sensitive to antibiotics, such as Cephamycin and Carbapenem. Bacteria that carry this enzyme can hydrolyze the corresponding antibiotics, leading to the failure of some treatments. Over the past several years, the dissemination of *E. coli* isolates produces ESBLs and pAmpC, which has been reported in different settings, including in food, food-producing animals, and different types of aquatic environments, especially wastewater (Diwan et al., 2012; Divesh et al., 2014; Warjri et al., 2015). In addition, wastewater can also provide favorable conditions for the growth of a diverse bacterial community, which constitutes a basis for the further selection and spread of antibiotic resistance (Ben Said et al., 2016).

Wastewater treatment plants (WTPs) are important reservoirs of human and animal micro-organisms that can enter into the environment again through the plant outlet, such as with water and food, and are likely to infect humans and animals. “The main transport pathways of antibiotics into the ambient environment are via WTPs, where they may be only partially eliminated” (Xu et al., 2007). So in this ecosystem, antibiotics in wastewater may exert a selective pressure that promotes the spread of the resistant microorganisms to other environments (Schlüter et al., 2007; Amos et al., 2014). In addition, WTPs’ wastewater contains a large number of bacteria, which is conducive to the bonding between bacteria, and this promotes transfer of multiple antibiotic resistance genes carried by movable elements. The discovery of R plasmid confirms that not only do the bacteria contain natural resistance genes, but also that they can acquire resistance to defend against survival pressures. This resistance is not only vertically transmitted, but it is also transmitted between species (i.e., horizontal transmission). The major factor in the spread of resistance is thought to be the ability of bacteria to acquire and transmit foreign genes through movable elements, such as plasmids and transposons (Mokracka et al., 2012).

The purpose of this study was to analyze the distribution of ESBLs-producing *E. coli* in municipal WTPs, to isolate ESBLs-producing *E. coli* strains, and then to elucidate the multiple antibiotic resistance linked transfer using a conjugative test. The resistant phenotypes and multiple antibiotic resistant genotypes were compared in transconjugants, donor and recipient strains. At last, we investigated the role of plasmids in the multiple antibiotic resistance transfer mechanism in *E. coli* by analyzing its full-length sequence.

## Materials and Methods

### WTPs and Sample Collection

The wastewater samples were taken from a municipal WTPs, located in Tai’an county, China, in September 2016. The WTPs employed an activated sludge process. The wastewater was taken from a hospital and a multi-species slaughterhouse. The samples used for research were taken from (i) raw wastewater in the primary sedimentation tank (intake), (ii) treated water (aeration tank), and (iii) final treated wastewater (outlet). In each sampling event, the samples were taken simultaneously from the three sites. The samples were collected in sterile containers at the depth of 0.3 m and the distance of 1 m from the side of the respective sampling sites as previously described (Mokracka et al., 2012). Each sample was refrigerated and then transported to the lab and analyzed within 12 h.

### Isolation and Identification of ESBLs-Producing *E. coli*

The isolation and the identification of *E. coli* were done following previously described methods (Mokracka et al., 2012). Briefly, the samples were diluted serially in 0.9% NaCl, inoculated onto Brilliance ^TM^ *E. coli*/Coliform Selective Agar (Oxoid) and incubated at 37°C for 24 h. Then the single colony was passaged three times for the further experiments. Identification of bacteria was done with API 20E kit (bioMerieux), dedicated to identifying *E. coli* and other Gram-negative bacteria using biochemical tests.

The suspected ESBLs-producing *E. coli* isolates were confirmed by phenotypic confirmatory tests using cefotaxime (30 ug), cefotaxime/clavulanic acid (30 ug/10 ug), ceftazidime (30 ug), and ceftazidime/clavulanic acid (30 ug/10 ug) (Kim et al., 2017; Zhang et al., 2017).

### Conjugation and Identification of Transconjugants

In order to prove the antibiotic resistance gene in *E. coli* has the ability to transfer *in vitro*, 50 ESBLs-producing *E. coli* strains were isolated from the WTPs, which were resistant to cefotaxime and sensitive to sodium azide. *E. coli* J53 was resistant to sodium azide and sensitive to most antibiotics, which was donated by Professor Yu-Song Yu from Zhejiang University School of Medicine. Conjugative testing was performed using the filter mating method (Wei et al., 2014; Knudsen et al., 2018). The suspected colonies were identified and the positive strains were passaged three times from the culture plates to a new antibiotics selective medium plate by scribing. They were then preserved in glycerol for subsequent experiments (Zhang, 2006; Knudsen et al., 2018).

### Detection of Antibiotic Susceptibility and Antibiotic Resistant Genotypes

Susceptibility analysis to 16 antibiotics Florfenicol (FFC), Sulfamethoxazole (SXT), Ampicillin (AMP), Aztreonam (AZT), Kanamycin (KAN), Cefalotin (KF), Cefepime (FEP), Norfloxacin (NOR), Streptomycin (STR), Ciprofloxacin (CIP), Imine imipenem (IPM), Chloramphenicol (C), Erythromycin (E) and Gentamycin (CN), Tetracycline (TE) was carried out by disk-diffusion method (Clinical and Laboratory Standards Institute [CLSI], 2013). *E. coli* ATCC 25922 was used as a reference strain (Silva et al., 2010). All screen-positive ESBLs-producing strains and transconjugants were from plasmids and genomic DNA extraction. They were then examined for the presence of *CTX-M*, *OXA*, *SHV*, *TEM*, *qnrA*, *qnrB*, and *qnrS* genes by multiplex PCR with the same method and primers as our earlier research, and the primers described in Table 1 (Li et al., 2017). DNA sequencing using purified PCR products was provided by ABI PRISM 3730XL Analyzer (Applied Biosystems, Foster City, CA, United States) in Shanghai Sangon Biotech, Co., Ltd., China. The database similarity searches for nucleotide sequences performed using the BLAST tool at the National Center for Biotechnology Information (NCBI) website^1^.

**Table 1:** Sequences of primers used for PCR.

Gene	Primer sequence (5′–3′)	Product length/bp
*bla*_SHV_	F: GGGTTATTCTTATTTGTCGCT	913
	R: GGGTTAGCGTTGCCAGTG	
*bla*_TEM_	F: GAGACAATAACCCTGGTAAATG	886
	R: AATGATTAATCAGTGAGGC	
*bla*_CTX-M_	F: AAGAAAAGTGAAAGCGAA	548
	R: GTGAAGTAAGTGACCAGAATC	
*qnrA*	F: TCAGCAAGAGGATTTCTCA	627
	R: GGCAGCACTATTACTCCCA	
*qnrB*	F: ATGACGCCATTACTGTATAA	562
	R: GATCGCAATGTGTGAAGTTT	
*qnrS*	F: ACCTTCACCGCTTGCACATT	576
	R: CCAGTGCTTCGAGAATCAGT	
*OXA*	F: CTGTTGTTTGGGTTTCGCAAG	591
	R: CTTGGCTTTTATGCTTGATC	


### Analysis of the Ligated Plasmids

After plasmid electrophoresis analysis, all plasmids were successfully extracted from all *CTX-M* and *TEM* gene-positive binders. Strains showed great variation in banding numbers and distance, containing 1 to 6 plasmids (∼2 to >120 kb). *E. coli* A8 showed only one about 83 kb plasmid carrying *CTX-M-15*, *TEM-1* and *qnrS* and therefore was used as an analysis target. Plasmid wwA8 was extracted with TIAGEN company plasmid extraction kit by following the instructions and was sent to Sangon company for analysis of the whole DNA sequence. After sequencing was completed, the open reading frame of the plasmid sequence was predicted using the Bacterial Annotation System and the result was confirmed with DNAMAN 5.2.10 software (BASys^2^; Van et al., 2005). Each predicted protein was compared to all protein databases using BlastP^3^. The gene sequence was further aligned with the GenBank database by BLAST, and the sequence homology plasmid resembled the reference plasmid^3^. *E. coli* strain PGR46 plasmid pPGRT46 (GenBank Accession No. KM023153.1) was used as a reference plasmid for WWA8 annotation. Plasmid maps were drawn using SnapGene Viewer 3.2.1.

## Results

### Distribution of ESBLs-Producing *E. coli*

Seventy *E. coli* strains were isolated from 80 wastewater samples with a separation rate of 87.5%. Among them, 25 out of 25 (100%) strains were isolated from intake, 30 out of 30 (100%) strains from aeration tank, and 15 out of 25 (60%) strains from outlet. ESBLs-producing strains could be identified according to the CLSI2009 standard, the ESBLs-producing strains were confirmed by phenotypic confirmation. A total of 50 ESBLs-producing isolates were obtained from 70 isolates of *E. coli*, with the isolation rate as high as 71.4%, of which 22 out of 25 (88%) were from water intakes, 20 out of 30 (66.7%) from aeration tanks and 8 out of 15 (53.3%) from water outlets.

### Identification of Conjugation

After the conjugative test using the filter mating method, the ERIC-PCR, and the selective plate assay, it was judged according to the conjugative screening test (Zhang, 2006). Fifty strains of ESBLs-producing resistance to Cefotaxime were used as donor bacteria, and 35 transconjugants were obtained successfully with the transfer rate as high as 70%.

### Resistant Phenotype of Donor Bacteria and Transconjugants

The resistant phenotypes of 35 transconjugants for 15 kinds of antibiotics compared to the donor strains were shown in Table 2. The results showed that all transconjugants and donor strains were capable of multiple antibiotic resistance for three or more antibiotics compared to recipient strain *E. coli* J53, which is sensitive to the above-mentioned 15 antibiotics. Both transconjugants and donors were capable of resistance to the AMP and KF at a rate of 100.00% (35/35). Among them, transconjugants had transferred STR, SXT, E, and KAN resistance compared to donors at a rate of 90.91% (20/22), 34.48% (10/29), 16.67% (2/12), and 22.22% (2/9). However, the capability of resistance to STR, SXT, E, and KAN in transconjugants broadened at a rate of 76.92% (10/13), 50.00% (3/6), 4.35% (1/23), and 7.69% (2/26). So transconjugants which had a narrowed antibiotic resistance spectrum, lost one or several antibiotic resistances which were present in the donor bacteria, or had a broadened antibiotic resistance spectrum and gained one or several antibiotic resistances which were not present in the donor bacteria. In a word, the antibiotic resistant spectrum of transconjugants narrowed after exposure to the donor bacteria at the rate of 94.29% (33/35) and broadened at the rate of 5.71% (2/35).

**Table 2:** Antibiotic resistance phenotypes of donor strains and transconjugants.

Transconjugant	Donor strains
AMP-KF-STR	AMP-KF-STR-C-FFC-CN-TE-KAN
AMP-AZT-KF-FEP-STR-E-TE	SXT-AMP-AZT-KF-FEP-STR-C-AZT-CIP-CN-TE-NOR
SXT-AMP-KF-FEP-STR-C-TE	AMP-KF-STR-C-CN-TE-KAN-FFC-IPM
SXT-AMP-AZT-KF-STR	AMP-AZT-KF-NOR-FEP-E-CIP-IPM
SXT-AMP-AZT-KAN-KF-STR-CIP-C	SXT-AMP-AZT-KAN-KF-CN-TE-FEP-E-IPM
SXT-AMP-AZT-KAN-KF-STR-E	SXT-AMP-AZT-KF-E-TE
SXT-AMP-AZT-KF-STR-C-TE	AMP-AZT-KF-TE-CN-NOR
SXT-AMP-AZT-KF-STR	SXT-AMP-AZT-KF-CN-TE-NOR-KAN-C-FFC
SXT-AMP-AZT-KF-STR	SXT-AMP-AZT-KF-STR-CN-TE-KAN-C-E-CIP-FFC
AMP-AZT-KF-STR	SXT-AMP-AZT-KAN-KF-STR-CN-TE-NOR-C-E-CIP-FFC
SXT-AMP-AZT-KF-STR	SXT-AMP-AZT-KF-CN-TE-FEP-C-E-IPM
SXT-AMP-AZT-KAN-KF-STR	SXT-AMP-AZT-KF-STR-TE-FEP
AMP-AZT-KF-STR-E	SXT-AMP-AZT-KF-STR-E-TE
SXT-AMP-AZT-KF-STR	SXT-AMP-AZT-KF-CN-TE-IPM
AMP-AZT-KAN-KF-STR	SXT-AMP-AZT-KAN-KF-STR-CN-TE
AMP-AZT-KF-FEP-STR	AMP-AZT-KF-STR-NOR-IPM-KAN
AMP-AZT-KF-STR	SXT-AMP-AZT-KF-STR-CN-NOR-CIP-IPM
SXT-AMP-AZT-KF-STR	SXT-AMP-AZT-KF-STR-CN-FEP
AMP-AZT–KF-STR	SXT-AMP-AZT-KF-STR-TE
AMP-AZT-KF-STR	SXT-AMP-AZT-KF-STR-CN-TE-NOR-C-CIP-FFC
AMP-KF-FEP–STR	SXT-AMP-AZT-KF-FEP-TE
AMP-AZT-KF-STR	SXT-AMP-AZT-KF-CN-TE
AMP-AZT-KF-FEP–STR	SXT-AMP-AZT-KF-FEP-STR-TE-E
AMP-AZT–KF-FEP-STR	SXT-AMP-AZT-KF-FEP-STR-IPM-TE
AMP-KF-FEP-STR	AMP-KF-STR-AZT-IPM
AMP-AZT-KF-STR	SXT-AMP-AZT-KF-FEP-E-IPM
AMP-AZT-KF-STR	SXT-AMP-AZT-KF-STR-TE-E-IPM
AMP-AZT-KF-FEP-STR	SXT-AMP-AZT-KF-FEP-STR-CN-TE-E-IPM
AMP–KF-STR	SXT-AMP-KF-AZT-STR-CN-TE-NOR-KAN-C-CIP-FFC-IPM
SXT-AMP-AZT-KF-FEP-STR	SXT-AMP-AZT-KF-STR-TE-IPM
SXT-AMP-AZT-KF-FEP	SXT-AMP-AZT-KF-FEP-IPM-TE
AMP-AZT-KF	SXT-AMP-AZT-KF-CIP-IPM-TE
AMP-AZT-KF	SXT-AMP-AZT-KF-E-IPM-TE-STR
AMP-AZT-KF	SXT-AMP-AZT-KF-FEP-TE-IPM
AMP-AZT-KF	SXT-AMP-AZT-KF-TE-STR-IPM


*NAL, nalidixic acid; E, erythromycin; CIP, ciprofloxacin; FFC, florfenicol; IPM, imipenem; AML, amoxicillin; SXT, cotrimoxazole; AMP, ampicillin; CN, gentamicin; TE, tetracycline; STR, streptomycin; NOR, norfloxacin; KAN, kanamycin; FEP, cefepime; KF, cefalotin; AZT, aztreonam; C, chloramphenicol; CTX, cefotaxime; CAZ, ceftazidime*.

### Antibiotic Resistant Genotypes of Donor Bacteria and Transconjugants

The resistant gene phenotypes of 35 transconjugants compared to its donor strains by PCR were shown in Table 3. The results showed that the *bla_TEM_* and *bla_CTX-M_* genes were all transferred successfully at the rate 100.00% (35/35). The *bla_SHV_* gene was transferred successfully at the rate 94.29% (33/35). However, only one strain of the *qnrS* gene was transferred at the rate of 4.17% (1/24). Blast comparison results showed that the gene subtype of the major resistance was *bla_TEM-1_*, *bla_SHV -11_* and *bla_CTX-M-15_*, and at the rate of 82.86% (29/35), 85.71% (30/35), and 85.71 (30/35), respectively.

**Table 3:** The multiple antibiotic resistant genotypes of 35 strains of donors and transconjugants.

Transconjugant	Donor strain
*bla*_TEM-135_–*bla_SHV -11_*–*bla*_CTX-M-15_	*bla*_TEM-135_–*bla*_SHV -11_–*bla*_CTX-M-15_–*qnrS*
*bla*_TEM-1_–*bla*_CTX-M-15_	*bla*_TEM-1_–*bla*_SHV -11_–*bla*_CTX-M-15_–*qnrS*
*bla*_TEM-135_–*bla*_SHV -11_–*bla*_CTX-M-15_	*bla*_TEM-135_–*bla*_SHV -11_–*bla*_CTX-M-15_–*qnrS*
*bla*_TEM-1_–bla_CTX-M-15_–*qnrS*	*bla*_TEM-1_–*bla*_SHV -11_–*bla*_CTX-M-15_–*qnrS*
*bla*_TEM-1_–*bla*_SHV -11_–*bla*_CTX-M-15_	*bla*_TEM-1_–*bla*_SHV -11_–*bla*_CTX-M-15_
*bla*_TEM-1_–*bla*_SHV -11_–*bla*_CTX-M-15_	*bla*_TEM-1_–*bla*_SHV -11_–*bla*_CTX-M-15_–*qnrS*
*bla*_TEM-1_–*bla*_SHV -11_–*bla*_CTX-M-15_	*bla*_TEM-1_–*bla*_SHV -11_–*bla*_CTX-M-15_–*qnrS*
*bla*_TEM-1_–*bla*_SHV -11_–*bla*_CTX-M-15_	*bla*_TEM-1_–*bla*_SHV -11_–*bla*_CTX-M-15_–*qnrS*
*bla*_TEM-1_–*bla*_SHV -40_–*bla*_CTX-M-15_	*bla*_TEM-1_–*bla*_SHV -40_–*bla*_CTX-M-15_–*qnrS*
*bla*_TEM-181_–*bla*_SHV -11_–*bla*_CTX-M-15_	*bla*_TEM-181_–*bla*_SHV -11_–*bla*_CTX-M-15_
*bla*_TEM-181_–*bla*_SHV -11_–*bla*_CTX-M-55_	*bla*_TEM-181_–*bla*_SHV -11_–*bla*_CTX-M-55_
*bla*_TEM-1_–*bla*_SHV -11_–*bla*_CTX-M-15_	*bla*_TEM-1_–*bla*_SHV -11_–*bla*_CTX-M-15_–*qnrS*
*bla*_TEM-1_–*bla*_SHV -11_–*bla*_CTX-M-15_	*bla*_TEM-1_–*bla*_SHV -11_–*bla*_CTX-M-15_–*qnrS*
*bla*_TEM-1_–*bla*_SHV -11_–*bla*_CTX-M-15_	*bla*_TEM-1_–*bla*_SHV -11_–*bla*_CTX-M-15_–*qnrS*
*bla*_TEM-1_–*bla*_SHV -56_–*bla*_CTX-M-15_	*bla*_TEM-1_–*bla*_SHV -6_–*bla*_CTX-M-15_–*qnrS*
*bla*_TEM-1_–*bla*_SHV -11_–*bla*_CTX-M-15_	*bla*_TEM-1_–*bla*_SHV -11_–*bla*_CTX-M-15_–*qnrB*
*bla*_TEM-1_–*bla*_SHV -11_–*bla*_CTX-M-15_	*bla*_TEM-1_–*bla*_SHV -11_–*bla*_CTX-M-15_
*bla*_TEM-181_–*bla*_SHV -40_–*bla*_CTX-M-55_	*bla*_TEM-181_–*bla*_SHV -40_–*bla*_CTX-M-55_–*qnrS*
*bla*_TEM-1_–*bla*_SHV -11_–*bla*_CTX-M-15_	*bla*_TEM-1_–*bla*_SHV -11_–*bla*_CTX-M-15_–*qnrS*
*bla*_TEM-1_–*bla*_SHV -11_–*bla*_CTX-M-15_	*bla*_TEM-1_–*bla*_SHV -11_–*bla*_CTX-M-15_–*qnrS*
*bla*_TEM-1_–*bla*_SHV -11_–*bla*_CTX-M-15_	*bla*_TEM-1_–*bla*_SHV -11_–*bla*_CTX-M-15_–*qnrS*
*bla*_TEM-1_–*bla*_SHV -11_–*bla*_CTX-M-15_	*bla*_TEM-1_–*bla*_SHV -11_–*bla*_CTX-M-15_–*qnrS*
*bla*_TEM-1_–*bla*_SHV -56_–*bla*_CTX-M-15_	*bla*_TEM-1_–*bla*_SHV -56_–*bla*_CTX-M-15_–*qnrS*
*bla*_TEM-1_–*bla*_SHV -11_–*bla*_CTX-M-15_	*bla*_TEM-1_–*bla*_SHV -11_–*bla*_CTX-M-15_–*qnrS*
*bla*_TEM-1_–*bla*_SHV -11_–*bla*_CTX-M-15_	*bla*_TEM-1_–*bla*_SHV -11_–*bla*_CTX-M-15_
*bla*_TEM-1_–*bla*_SHV -11_–*bla*_CTX-M-55_	*bla*_TEM-1_–*bla*_SHV -11_–*bla*_CTX-M-55_
*bla*_TEM-1_–*bla*_SHV -11_–*bla*_CTX-M-15_	*bla*_TEM-1_–*bla*_SHV -11_–*bla*_CTX-M-15_–*qnrS*
*bla*_TEM-1_–*bla*_SHV -11_–*bla*_CTX-M-15_	*bla*_TEM-1_–*bla*_SHV -11_–*bla*_CTX-M-15_–*qnrS*
*bla*_TEM-1_–*bla*_SHV -11_–*bla*_CTX-M-55_	*bla*_TEM-1_–*bla*_SHV -11_–*bla*_CTX-M-55_
*bla*_TEM-116_–*bla*_SHV -11_–*bla*_CTX-M-15_	*bla*_TEM-116_–*bla*_SHV -11_–*bla*_CTX-M-15_–*qnrS*
*bla*_TEM-1_–*bla*_SHV -79_–*bla*_CTX-M-15_	*bla*_TEM-1_–*bla*_SHV -79_–*bla*_CTX-M-15_–*qnrS*
*bla*_TEM-1_–*bla*_SHV -11_–*bla*_CTX-M-55_	*bla*_TEM-1_–*bla*_SHV -11_–*bla*_CTX-M-55_–*qnrB*
*bla*_TEM-1_–*bla*_SHV -11_–*bla*_CTX-M-15_	*bla*_TEM-1_–*bla*_SHV -11_–*bla*_CTX-M-15_
*bla*_TEM-1_–*bla*_SHV -11_–*bla*_CTX-M-15_	*bla*_TEM-1_–*bla*_SHV -11_–*bla*_CTX-M-15_–*qnrS*
*bla*_TEM-1_–*bla*_SHV -11_–*bla*_CTX-M-15_	*bla*_TEM-1_–*bla*_SHV -11_–*bla*_CTX-M-15_


### Analysis of the Transferred Plasmid

**FIGURE 1:**
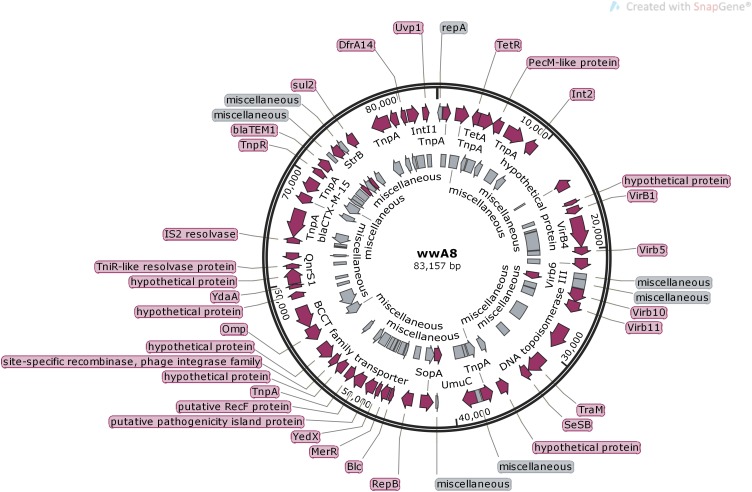
Genetic map of the plasmid wwA8.

A plasmid harbored in *E. coli* A8 was named wwA8 (GenBank MG773378), and its pattern map drawing with the whole DNA sequence was displayed in Figure 1. Plasmid wwA8 is a closed-loop DNA molecule with 83157 bp and GC content at the rate of 52.74%. The plasmid wwA8 contains 45 predicted genes (Table 4), carries three known antibiotic resistance genes, *bla_CTX-M-15_*, *bla_TEM-1_*, *qnrS1*, which can be transferred in *E. coli in vitro*. The sequence analyzing results of the plasmid showed that *E. coli* isolated from wastewater had the proficiency of resistance genes transferring. The basic structure of plasmid wwA8 is very homologous to plasmid IpPGRT46 (GenBank KM023153.1).

**Table 4:** Open reading frames identified in wwA8.

Gene name	Nucleotide position	Function encoded
*repA*	72–487	IncN replicase gene distrupted by insertion of IS26
*TnpA*	539–1255	Transposase IS26
*TnpA*	1720–2940	Transposase for transposon Tn1721
*TetR*	3272–3949	Tetracycline repressor protein
*TetA*	3953–5227	Tetracycline efflux protein
*Int2*	8775–9752	Integrase/recombinase
*VirB1*	16090–16752	Type IV secretory pathway VirB1 component
*VirB4*	17072–19822	Type IV secretion system protein virB4
*Virb5*	19841–20557	P-type DNA transfer protein VirB5
*Virb6*	20871–21878	VirB6 plasmid conjugal transfer protein
*Virb8*	22088–22774	Type IV secretion system protein virB8
*Virb9*	22767–23660	P-type conjugative transfer protein VirB9
*Virb10*	23657–24853	Type IV secretion system protein virB10
*Virb11*	24857–25906	P-type DNA transfer ATPase VirB11
*DNA topoisomerase III*	27345–29546	DNA topoisomerase III family protein
*TraM*	30755–32551	Mobilization protein
*SeSB*	32566–33312	Mobilization protein
*Hypothetical protein*	34775–35740	Antirestriction protein
*TnpA*	36357–37415	Transposase of ISL3
*UmuC*	37941–39212	UV protection
*SopB*	40935–41906	Plasmid-partitioning protein
*SopA*	41906–43081	Plasmid partition protein SopA
*RepB*	43813–44823	Initiator replicase protein FIB-like replicon
*Blc*	45614–45973	Outer membrane lipoprotein precursor
*MerR*	46076–46801	Transcriptional regulator MerR
*YedX*	46901–47311	Hydroxyisourate hydrolase
*TnpA*	49670–50386	Transposase of IS26
*Omp*	54416–55708	Putative membrane protein
*YdaA*	59566–59925	Resolvase-like protein, YdaA
*QnrS1*	62133–62789	Quinolone resistance gene
*TnpA*	64242–66791	Transposase for transposon Tn3
*blaCTX-M-15*	67430–68305	Beta-lactamase enzyme family
*TnpA*	68561–69823	ISEcp1 transposase
*tnpA*	70005–70382	Fragment
*blaTEM1*	71127–71987	Beta lactamase TEM-1
*TnpA*	72197–72736	Transposase of ISVsa3
*StrB*	72708–73544	Streptomycin resistance protein B
*StrA*	73544–74035	Aminoglycoside phosphotransferase
*sul2*	74408–75223	Dihydropteroate synthase
*TnpA*	76994–78700	Tn3 family transposase
*TnpA*	78812–79528	Transposase of IS26
*DfrA14*	79842–80324	Dihydrofolate reductase DfrA14
*IntI1*	80471–81484	Class 1 integron integrase
*TnpM*	81423–81737	Transposon Tn21 modulator protein
*Uvp1*	81877–82446	Resolvase of the R46 plasmid


## Discussion

*Escherichia coli* are important opportunistic pathogens that cause urinary tract infections and sepsis in animals and humans (Lewis et al., 2007). The prevalence of multiple antibiotic resistant Enterobacteriaceae in the world has been increasing in recent decades. β-lactams and fluoroquinolones have been selected as important therapeutic agents. The selective pressure created by the abuse of these agents has led to the development of multiple antibiotic resistant bacteria. One of the mechanisms by which multiple antibiotic resistant bacteria are produced is the production of plasmid-mediated ESBLs which hydrolyze β-lactam (Cantón et al., 2008). ESBLs can hydrolyze β-lactam and propagate through bacteria in a plasmid-mediated manner, which is one of the main reasons for Gram-negative bacilli resistance. The gene coding for ESBLs is located on the plasmid, which has many genotypes such as *bla_CTX-M_*, *bla_SHV_*, *bla_TEM_* and *OXA* types. Bacterial genes encoding ESBLs are often located on the same plasmid with other antibiotic resistance genes, leading to multiple bacterial resistances, causing great difficulties in clinical treatment of infectious diseases (Ben-Shahar et al., 2012).

The genes encoding ESBLs are located on the plasmids. There is diversity in genotypes of ESBLs including *bla_CTX-M_*, *bla_SHV_*, *bla_TEM_*, *OXA*, etc. Due to the different geographical and antibiotic habits, the prevalence of genotypes in different countries, regions, and environments varies (Fabre et al., 2009). Animal-derived ESBLs-producing *E. coli* has been reported (Alexy et al., 2006), but less ESBLs-producing *E. coli* is reported in wastewater. In this paper, ESBLs-producing *E. coli* were isolated from WTPs, and then *E. coli* J53 was as recipient bacteria performed plasmid conjugation, the multiple antibiotic resistance phenotype and the multiple antibiotic resistant genotypes test were carried out. One of the plasmids in transconjugants was sequenced to detect the transfer of the plasmids in the bacteria. In this experiment, 50 isolates of ESBLs-producing *E. coli* were isolated from 80 wastewater samples and the isolation rate was very high. Therefore, ESBLs-producing *E. coli* has been widespread in the environment. Among them, the outlet ESBLs-producing *E. coli* separation rate is 32%, and at the intake the separation rate is 88%. Although WTPs can significantly reduce the microbial load in water, it cannot completely eliminate antibiotic resistance bacteria. On the contrary, these selective pressures increase the resistance of certain bacteria. The ESBLs-producing *E. coli* in the outlet water cannot be completely eliminated. It will enter the local environment, resulting in the spread of resistant bacteria. On the other hand, untreated wastewater overflow into the surface during rainstorms may be one of the sources of ESBLs-producing *E. coli* (Diallo et al., 2013).

In this experiment, 50 ESBLs-producing *E. coli* strains were isolated from municipal WTPs in Tai’an City, 35 strains were successfully transferred. The detection of antibiotic resistant ESBLs-producing genes showed that three genotypes of *blaCTX-M*, *bla_SHV_* and *bla_TEM_* were detected, which was consistent with the previous study (Cohen Stuart et al., 2010; Sima et al., 2016). No *OXA* genotype was detected in this study and a small amount of the fluoroquinolone resistance gene was detected. The *bla_TEM_* and *bla_CTX-M_* genes were transferred successfully in all strains, except for the *bla_SHV_* only in which only one strain transferred successfully. With the increasing use of β-lactam antibiotics, especially the third-generation cephalosporins, it is important to monitor the production of *bla_CTX-M_*, *bla_SHV_*, and *bla_TEM_* strains. In particular, it is important to monitor the surveillance of *bla_CTX-M_*, *bla_SHV_*, *bla_TEM_* genotype transmission in order to provide a reliable basis for clinical use of antibiotics.

The mechanism of bacterial resistance is quite complex. However, great progress has been made in the research of this topic. In particular, research of the R plasmid confirms that the genetic material contains the natural resistance gene in bacteria. Acquired antibacterial resistance is gained via selective stress. Conjugation is the most common way genetic information is transferred and plays a very important role in the spread of multiple antibiotic resistance genes. 35 conjugations of *E. coli* J53 were finally obtained, and the success rate of conjugation was as high as 70%. The results show that under certain selective pressures, the plasmid is very easily transferred between *E. coli*, leading to the spread of antibiotic resistance, which is very harmful to clinical treatment (Cavaco et al., 2007).

The antibiotic resistant spectrum of transconjugants narrowed compared to the donor bacteria at the rate of 94.29% (33/35). This could mean that the antibiotic resistance gene may be located in the movable elements such as plasmids rather than the genomes (Park et al., 2017), or that different strains carry different plasmids, some of which are not compatible. However, the antibiotic resistance spectrum of transconjugants broadened compared to donor bacteria at the rate of 5.71% (2/35). In addition, transconjugants which lost one or more antibiotic resistances also added one or more antibiotic resistances at the rate of 48.6%. These are why antibiotics should be used with caution so as not to cause an increase in antibiotic resistance. At the same time, there was a significant increase in the resistance to STR, which may be caused by the enhanced expression of *aadA1* and *aadA2* gene cassettes located on the transferred plasmid, showing resistances that are not in donor bacteria (Zhao et al., 2011). The transfer rate of AMP and KF in ESBLs-producing *E. coli* was 100%. This proved that the plasmids in *E. coli* play an important role in the multiple antibiotic resistant transfer.

## Conclusion

This study shows that *E. coli* isolated from wastewater was capable of resistance gene transfer and of producing antibiotic resistance phenotypes. The resistance genes are located on plasmids which have the ability to transfer *in vitro*, and the plasmids in *E. coli* play an important role in the multiple antibiotic resistance linked transfer.

## Author Contributions

QL performed the experiments and analyzed the data. WC drafted the manuscript. HZ and DH collected wastewater samples and some data. XW designed and supervised the study and performed manuscript editing.

## Conflict of Interest Statement

The authors declare that the research was conducted in the absence of any commercial or financial relationships that could be construed as a potential conflict of interest.

## Footnotes

1 http://www.ncbi.nlm.nih.gov/BLAST

2 http://wishart.biology.ualberta.ca/basys/cgi/submit.pl

3 http://blast.ncbi.nlm.nih.gov/Blast.cgi
